# Community Conversations: Stakeholder-Identified Research Priorities to Foster Community Participation for Individuals With Intellectual and Developmental Disability

**DOI:** 10.3389/fresc.2022.873415

**Published:** 2022-06-21

**Authors:** Roxanna M. Bendixen, Teal Benevides, Roger Ideishi, Robert Smythe, Joshua Taylor, Caroline Umeda, Cheryl Kerfeld, Tracy Jirikowic

**Affiliations:** ^1^Department of Occupational Therapy, University of Pittsburgh, Pittsburgh, PA, United States; ^2^Department of Occupational Therapy, Augusta University, Augusta, GA, United States; ^3^Department of Occupational Therapy, George Washington University, Washington, DC, United States; ^4^Philadelphia, PA, United States; ^5^College of Education and Human Development, University of Maine, Orono, ME, United States; ^6^Department of Occupational Therapy, Dominican University of California, San Rafael, CA, United States; ^7^Division of Physical Therapy, Seattle Public Schools, Seattle, WA, United States; ^8^Department of Occupational Therapy, University of Washington, Seattle, WA, United States

**Keywords:** community participation, intellectual and developmental disability, stakeholder engagement, research capacity building, community engagement, inclusion

## Abstract

To identify future research priorities and meaningful outcomes focused on community-level interventions for children and youth with intellectual and developmental disabilities and families, a group underrepresented in research, we established a diverse patient-centered outcomes research (PCOR) community. We focused on engaging regionally and nationally-diverse stakeholders—individuals, families, healthcare professionals, community, and policy experts—in research development activities that would build partnerships and research capacity. This community of stakeholders also represented the matrix of systems, services, and programs that people frequent in their communities (e.g., cultural arts, worship, sports and recreation, and transportation). We present the engagement process and methods for including individuals with intellectual and developmental disabilities as stakeholders in research planning and processes. The results of planning, completing, and evaluating three face-to-face research capacity-building meetings and their subsequent stakeholder engagement activities include: (1) individuals with intellectual and developmental disabilities and their families clearly expressed a desire to be included and to feel good about their participation in community settings, (2) many of our stakeholders wanted action and change to happen in their communities now, and often did not realize or understand that research takes time, (3) organizations expressed a need for mentoring related to best practices for access and inclusive programming. Overarching issues around societal inclusion, equal opportunities, and life chances for individuals with intellectual and developmental disabilities and their families were front and center across communities and multi-stakeholder groups, and achieving change remains valued and a high priority.

## Introduction

Individuals with intellectual and developmental disabilities and their families often face social and physical environmental barriers within their communities that contribute to economic disparities and inequities in health, well-being, and quality of life (1, 2). For example, caregivers often report that those they care for on the autism spectrum have difficulty participating in the community due to social expectations and stigma (3). Research has determined that individuals with intellectual and developmental disabilities are often socially isolated (4, 5), have low levels of participation in competitive integrated employment (6, 7), have higher rates of sedentary behavior and obesity compared to individuals without intellectual and developmental disabilities (8, 9), and often have co-occurring mental health and psychiatric disorders (1, 2). Family members of individuals with intellectual and developmental disabilities report that barriers in physical and structural environments limit their ability to access community buildings, contributing to reduced participation (10), and increased social isolation (3, 11–13). Notably, social and environmental barriers that diminish community participation increase the risk of comorbid conditions and may lead to poorer physical and mental health outcomes (1, 2, 9, 14).

Meaningful participation in one's community contributes to physical and psychosocial health and well-being and is associated with improved health and social outcomes across the lifespan (5, 15–17). Modifying key factors in the physical or social environments are crucial to improving health outcomes of people with disabilities (18–20). Indeed, multiple aspects of the social and physical environment can be modified to provide support for children with intellectual and developmental disabilities to engage in community experiences that promote health and prepare them for the future (10, 21). Here we may consider a child's capacity for engaging with friends on a community playground, but the environment restricts the child's participation through lack of adaptive playground equipment or the placement of adaptive playground equipment too far away from a child's peers for social engagement. Although literature links community participation to health outcomes in children and youth with intellectual and developmental disabilities, most available research is correlational rather than causal (22). Further research is needed to assess the contributions of meaningful community-level interventions to health, well-being, and quality of life, and address those contributions in relation to community participation.

Recognizing the disparities in community participation and reduced health outcomes for children and youth with intellectual and developmental disabilities and their families (1, 4, 9), we created a patient-centered outcomes research (PCOR) community with a focus on co-creating research (research development) with an under-represented group. We sought to identify research priorities, methods, and meaningful outcomes to build a foundation for future research partnerships that develop and evaluate community-level interventions. For the purpose of this paper, we define community-level interventions as organizational practices that aim to reduce environmental barriers to participation that are either social (e.g., staff and public attitudes) or physical (e.g., levels of sensory stimulation). Community-level interventions are, by nature, designed to strengthen the health and welfare of the communities in which they are implemented. Such interventions enhance the capacity of community organization practices and programs, facilitate opportunities for individuals with intellectual and developmental disabilities and their families to participate in social activities, and establish health-promoting behaviors. For our team, community-level interventions also refer to practices that are rooted in models that promote participation (21, 23), are guided by healthcare professionals (such as occupational therapists, physical therapists, and speech and language pathologists), and are implemented in partnership with key organizations and community-stakeholders (24). Importantly, community-level interventions may differ between communities due to factors that include context and available resources. For example, community-level interventions may include organization-wide training for staff to improve attitudes and beliefs and/or the use of modifications to the physical environment to reduce sensory stimulation.

Our project was focused on identifying what constitutes community participation from the perspective of our PCOR community and gathering information from our stakeholders to more clearly understand their lived experiences and perspectives on involvement in life situations within their communities. Hence, to frame our conversations with stakeholders, we used the definition of participation as “involvement in a life situation” provided by the World Health Organization *International Classification of Functioning, Disability and Health* (ICF) (25). The ICF considers participation a component of health, and literature clearly states that to promote health we must address particular barriers that constrain community participation (20, 26). As our community conversations emerged, there was a lean toward social participation in the community in lieu of work or vocational activities (14, 27). An additional focus was to determine ways to measure the outcomes resulting from community participation that our stakeholders determined to be most important.

We were committed to the idea that, in order to fully plan and assess community interventions and meaningful programs, it was especially crucial to include individuals with intellectual and developmental disabilities and their families in the development of our research and intervention initiatives. Importantly, individuals with intellectual and developmental disabilities have been vastly under-represented as stakeholders in research (28), and their roles in the research process have usually been limited to that of research participants, rather than as co-creators or drivers of research priorities. While input from a range of community stakeholders is essential to determine short- and longer-term impact and patient-centered outcomes, the process must begin with individuals with intellectual and developmental disabilities and their families providing meaningful input to the research process (28, 29). Therefore, our collaborators included individuals with intellectual and developmental disabilities and their families, as well as other stakeholders who represented the community systems, services, and non-disability focused programs that people frequent in their communities (e.g., cultural arts, places of worship, sports and recreation, transportation services).

This paper describes our stakeholder engagement process and the outcomes that resulted from planning, implementing, and evaluating face-to-face research capacity building meetings in three U.S. regions (Northeast, Northwest, South) and their subsequent stakeholder engagement activities. Our overarching objectives were to (1) create a regional and national network of stakeholders to inform and partner in research, and (2) develop research priorities for community interventions and potential outcomes related to health and well-being for individuals with intellectual and developmental disabilities and their families.

## Methods

### Design

Our project focused on engaging regionally and nationally-diverse stakeholders—individuals, families, healthcare professionals, community, and policy experts—in ***research development*** activities that would build partnerships and research capacity. To build partnerships and research capacity, we brought stakeholders together to engage with each other and learn more about the obstacles that inhibit individuals, organizations, and communities from realizing the goal of inclusion (i.e., community participation). Our stakeholders informed our research priorities and desired patient-centered outcomes as they relate to community participation as a determinant of health, well-being, and quality of life.

### Ethics

Human subjects applications were submitted by the respective institutions representing each of the regional meetings. All the project activities and key information gathered were consistent among each regional site, but the IRB review process differed at each institution. Two institutions deemed the project exempt from human subjects research but required that permission be obtained from participants to audio record and take pictures at the meetings. In the case of the region where informed consent was required, the meeting participants were provided with an electronic document to review prior to the meeting. We then obtained written informed consent prior to the start of the meeting for all meeting participants.

### Developing a Regional and National Network

To achieve the objective of creating a regional and national network of stakeholders, the project leads and team members in our three respective regions (Northeast, Northwest, South) identified and enlisted Engagement Coordinators, Organization Partners, and Advisory Board Members. Depending on the community, our broader pool of stakeholders (meeting participants) were identified and involved in different ways. Some stakeholders came to us through established relationships and were engaged as part of the project application, while some stakeholders were identified through community outreach and word of mouth after the project began.

Initially, we engaged 12 stakeholders as our Advisory Board. The Board represented the diverse network of individuals and organizations across our three U.S. community regions with vested interest in our research development activities. The Advisory Board included:

- Individuals with intellectual and developmental disabilities
- Parents/caregivers and family members
- Leaders from community organizations (cultural arts, public transportation, education and vocational development)
- Healthcare professionals (occupational therapists, physical therapists, speech and language pathologists)
- Neighborhood and public transit providers
- Researchers
- Legal, policy, and advocacy experts

The Advisory Board was instrumental in vetting meeting agendas and proposed outcomes, as well as reviewing resources and materials created for each of our three regional research capacity-building meetings.

### Regional Engagement on Priority Areas for Research

We held three regional day-long, face-to-face research capacity building meetings with an extensive network of stakeholders in the Northeast (Philadelphia, PA), the Northwest (Seattle, WA), and the South (Augusta, GA). At each meeting, in addition to our regional Advisory Board members and engagement coordinators, we invited other local and regional stakeholders to participate; ~30 stakeholders attended each gathering. We intentionally limited the number of participants to ensure that meetings allowed for meaningful sharing in both large group discussions and small group breakout sessions. Meeting agendas were carefully developed, vetted by our Advisory Board, and emailed prior to the meeting date to give all participants time to review information about the meetings' purpose and goals, and to consider any questions they might have. As this project evolved, we created and recorded a pre-meeting video to accompany the written agenda for the third meeting in Augusta in response to feedback from previous meetings about ways to enhance communication with stakeholders.

The structure of each regional meeting was consistent: in the first part of the day, a large group discussion used open-ended questions for discovery of key facilitators and barriers of community participation in that region (Table 1). The main lines of questioning were broad, and we gave respondents the time and space to talk about what is important to them regarding community participation. The lead engagement coordinator used the following agenda items to jump-start the large group discussions before guiding participants through a process that elicited potential subjects for small group break-out discussions.

**Table 1:** Sample questions for the large group discussions.

What does participation mean to you?
What do you want? What is your wish list? What is your top priority?
What are things that people do that show that they have included everyone (i.e., what's working?)
What are things that people do that show they have not included everyone (i.e. what's not working)?
How should we stay in touch with each other, and with other interested people?

Following a break, we held up to four small, 90-min working group discussions, each facilitated by project team members. In all regional meetings, these small breakout groups identified and discussed the topics of most interest and importance:

1) developing innovative ways to represent and engage all relevant stakeholder groups, emphasizing ways to give voice to children and youth with intellectual and developmental disabilities and their families as stakeholders;

2) identifying ways to engage and communicate with diverse stakeholders in other regions of the United States, along with ways to sustain these relationships and activities;

3) determining meaningful and multi-level person-centered outcomes and how we can effectively measure these outcomes (i.e., community participation); and

4) defining community-level interventions and components for future research, including comparative effectiveness research.

The project team and our lead engagement coordinator gathered qualitative information from stakeholders during every large and small working group discussion. Project team members collected field notes using several methods. During the large group morning meeting, the lead engagement coordinator used large note pads to document stakeholders' comments and quotes. The pages from these note pads were posted on the walls throughout the day so stakeholders could refer to them and offer follow-up comments to ensure that the information collected and recorded was accurate and relevant to our stakeholders. Additional field notes were gathered through written recording of thoughts/ideas and important key words, actual quotes captured during our discussions, and visual capture of diagrams and drawings. Multiple writers recorded notes using the verbatim principle as often as possible to accurately record what was taking place and being said in discussions, thereby minimizing stereotyping or inappropriately interpreting events. The use of both large and small group discussions allowed us to leverage stakeholders' experience and relationships and facilitate information sharing and consensus building.

**Figure 1:**
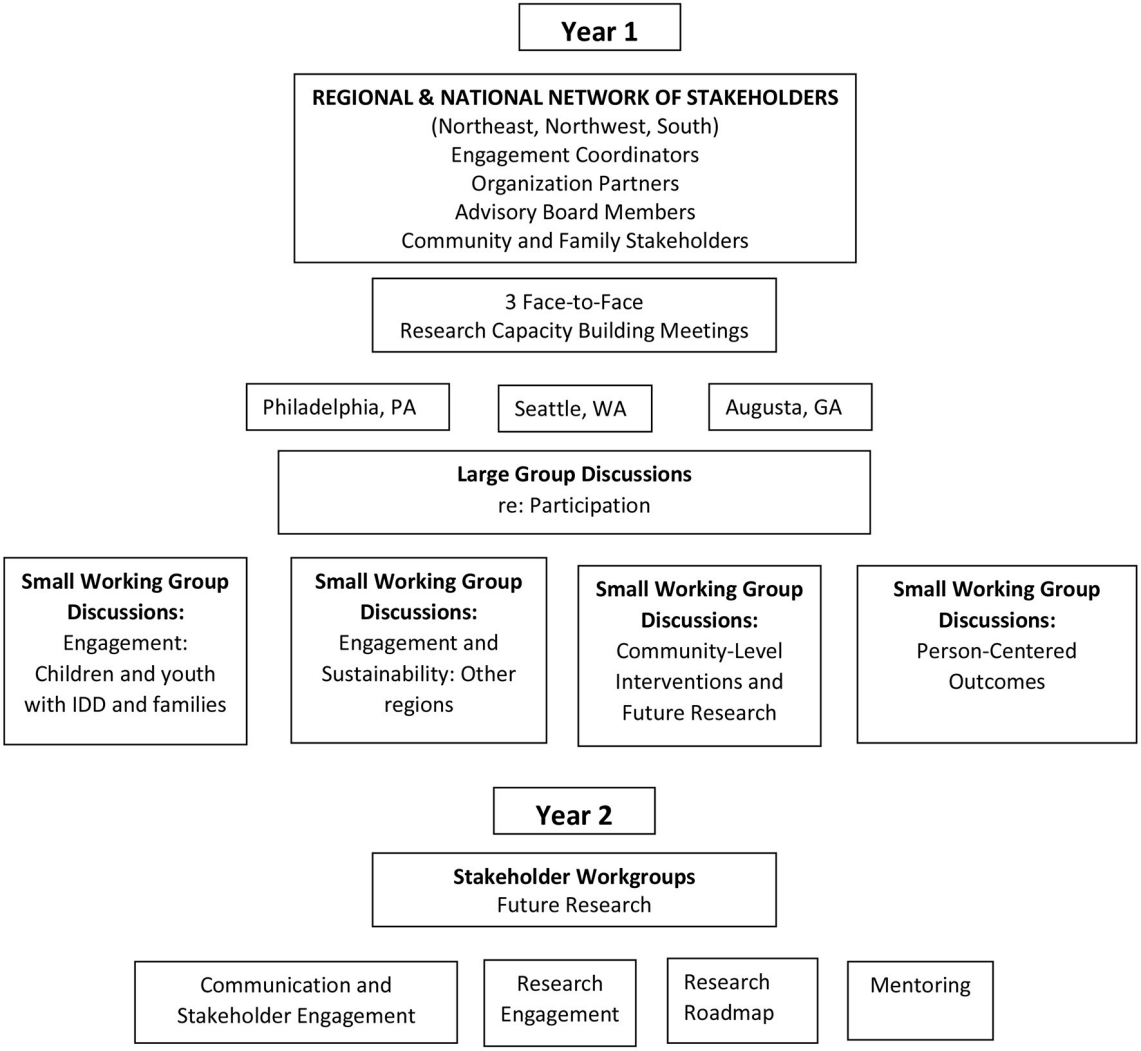
Representation of methodology for research capacity building face-to-face meetings.

Following each of the regional meetings, we summarized the gathered information using thematic analysis to identify, accumulate, and codify the input of each of our stakeholders: individuals with intellectual and developmental disabilities, their family members, and varied healthcare providers and community leaders. The use of field notes is appropriate for thematic analysis and was strengthened using multiple strategies for their collection (30). We then sent a summary of themes and key meeting outcomes—using an online survey platform—to each stakeholder to validate our initial meeting results (member-checking) and solicit additional thoughts and opinions for the purpose of consensus on the most important research questions and meaningful outcomes to measure. This gave stakeholders an equal opportunity to contribute by giving them time to reflect on the themes and meeting outcomes without the time pressure of a 1-day meeting. The online survey focused on prioritizing the action items that resulted from the large-group sessions and small working-group breakouts. The results from the survey were reported to all stakeholders. Figure 1 presents a graphical representation of the methodology.

## Results

Our multi-stakeholder groups represented a diverse set of individuals and organizational leaders from local communities. Table 2 describes each of the key stakeholder roles involved in this project.

**Table 2:** Key stakeholder roles.

**Stakeholder**	**Description**
Lead engagement coordinator (*n* = 1)	This person was very important to the project at all levels by identifying key questions to ask stakeholders, facilitating meeting discussions, and establishing rapport and trust. The lead engagement coordinator, as a disability self-advocate, attended team conference calls throughout the project, provided suggestions for plain language communication with our stakeholders during all phases of the project, communicated with stakeholders through video follow-up, and helped with the development of stakeholder training videos.
Advisory board members (*n* = 12)	Members of our advisory board represented all three regions and all stakeholder groups (e.g., self-advocates, parents, community members, clinicians, researchers, and administrators from partner organizations). Advisory board members were brought together to confirm meeting agenda items and results.
Regional engagement coordinators (*n* = 2)	Regional coordinators helped support local engagement with community members in each region and represented self-advocate and parent roles. Primary activities were assisting with recruitment of community members, attending regional meetings, helping with interpretation of meeting information, and reviewing project deliverables.
Organizational partners (*n* = 4)	The project partners' main contribution was to represent their organization's perspective at regional meetings and on the advisory board. The project partners represented organizations that have active inclusive programming and resources.
Regional meeting attendees (*n* = 93)	Ninety-three stakeholder participants attended three regional meetings.
Workgroup members (*n* = 37)	In Year 2, we engaged a subset of meeting attendees who continued to participate in the project to support workgroup activities.

All three regional face-to-face meetings were held in Year 1 of the 2-year project. The regional meetings were carried out in Philadelphia, PA (January 12, 2018: *n* = 31 participants), Seattle, WA (March 23, 2018: *n* = 28 participants), and Augusta, GA (July 13, 2018: *n* = 34 participants). A total of 93 stakeholders attended the meetings; meeting attendees represented the following stakeholder groups: self-advocates, family members of individuals with intellectual and developmental disability, researchers, community clinicians (e.g., occupational therapists, physical therapists) and members of community organizations (e.g., administrators, staff). Our stakeholders represented broad sectors of the community that included public transportation, employment services, cultural arts, libraries, faith-based organizations, sports and recreation and advocacy and support groups. Many attendees identified as having multiple roles (e.g., self-advocate and representative from a community organization, parent and representative from a community organization, research and practicing clinician). Our results focus on the views and perspectives of all stakeholders based on the specific discussion topic, not the specific stakeholder role.

### Stakeholder Identified Priorities

Each of our three community conversations (large and small groups) focused on what participation means, why participation is important, how communities and organizations can be more inclusive and supportive of participation, and what outcomes are important to measure to show change. Qualitative data were analyzed for content and themes. In order to ensure we were addressing themes essential to our stakeholders; we used an online follow-up survey focused on prioritizing the action items that resulted from the large-group sessions and small working-group breakouts. Results established consensus about the most important research priorities and the most meaningful outcomes to consider. This process identified six research priorities, with the first one serving as an overarching theme for the other five (Table 3).

**Table 3:** Stakeholder research priorities.

**Priority**	**Description**
Measuring success	When community organizations use so-called “best practices” for inclusive participation, how do we measure success?
Mentoring for organizations	When an organization wants to change and begin including individuals with ID/DD and families in their programs, how does working with a mentor support that change?
Trained transportation	If transportation partners, like bus drivers or Uber drivers, were trained to support individuals with ID/DD and families, would that make participating in the community easier?
Information access	How would getting information through technology, like smart phones and internet sites such as Facebook, support participating in the community?
Brand promise	Would community participation increase if there were a logo or brand that described an organization's ability to support the needs of individuals with ID/DD?
Advance support	To make community participation less stressful or more engaging for those with ID/DD, what types of support do organizations need to provide in advance?

The following describes discussions that occurred during our small working-group breakout sessions and what stakeholders told us were the important outcomes to measure in relation to the topic of “participation equity (community participation) and health and well-being for individuals with intellectual and developmental disabilities” (Table 4).

**Table 4:** Meaningful outcomes to measure in relation to community participation.

**Outcome**	**Description**
Social engagement	…in peer relationships and in friendships in school and/or the community.
Feelings of belonging	…to a larger group or community.
Community connectedness	…feeling like it is “worth it” to get out of the house and take part in an event or activity.
Excitement and motivation	…looking forward to and/or preparing for an event.
Resilience	…an individual, caregiver, or family's perseverance, tenacity, fortitude, or willingness to try something again.
Cheerfulness or happiness	…positive feelings about going out and/or doing the activity.
Feeling in control	…about going out and doing activities in the community
Taking part in related activities	…doing other activities on an outing such as going to a store, going to a park, or having a meal in a restaurant.
Social media connections and relationships	…which may be initiated and developed during and after engagement in the community.
Feelings of safety	…before, during, and after an event or activity.

These themes reflect broader, overarching issues around societal inclusion, equal opportunities, and life chances for individuals with intellectual and developmental disabilities and their families. These issues were very much front and center across communities and multi-stakeholder groups, and achieving change remains valued and a high priority. Community-level interventions were generally perceived as positively impacting individuals with intellectual and developmental disabilities, families, and community stakeholders by helping individuals and families feel safe and welcome, giving people opportunities and choices in decision-making, and facilitating successful participation in chosen community activities (18, 19).

Driven by the topics of interest for future research identified by stakeholders at the regional meetings and the desire for actionable next steps, we established four workgroups in Year 2 of the project. Through a follow-up email invitation, we invited all meeting stakeholders to participate in one or more workgroups. Thirty-seven stakeholders, or approximately one-third of the participants at all three regional meetings, stayed involved with the project in Year 2. Table 5 lists the workgroups and presents the breakdown of participants by region. Because some participants were assigned to multiple workgroups, the number of total participants by region is lower.

**Table 5:** Stakeholder workgroups and participants by region.

**Workgroup**	**Northeast**	**Northwest**	**South**	**Total**
Communication and engagement	1	6	5	12
Research engagement	2	4	-	6
Research roadmap	6	5	-	11
Mentoring	2	3	6	11

*Workgroup totals do not include project leads*.

Table 6 shows the goals of each workgroup that were co-created within the context of stakeholder input, and broader project aims and resources. Workgroup engagement and communication during Year 2 occurred through virtual meetings and email communication.

**Table 6:** Workgroup goals.

**Goals**	**Description**
Communication and engagement	• Develop communication guidance and customizable “template” for diverse community programs to meet regional needs. • Outline ways to expand and sustain regional groups; facilitate national network connections.
Research engagement	• Review engagement literature; existing training modules. • Create stakeholder engagement training modules. • Provide guidance to include individuals with ID/DD as stakeholders.
Research roadmap	• Refine meeting priorities into research questions. • Identify research outcomes (individual, family, organization-level). • Identify funding sources/write research grant(s).
Mentoring network	• Coordinate national mentoring network. • Establish efficient and accessible ways to share resources.

## Discussion

Throughout the course of this 2-year project, we engaged multiple stakeholder groups across three U.S. regions. While current research suggests that social inclusion and community participation is essential to enhancing a person's quality of life, much of this research speaks to stakeholder *derived* needs, but not stakeholder *driven* needs (5, 31, 32). For this project, our goal was to partner with stakeholders to co-create and validate research priorities before the research question(s) are generated. We experienced successes and challenges in our engagement process and built consensus on the most meaningful patient-centered research priorities and outcomes. Herein we summarize key engagement lessons learned to support ongoing and future research partnerships. In addition, we provide further information that will drive future intervention research around community participation as a health determinant for individuals with intellectual and developmental disabilities.

### What We Learned About Future Research Priorities and Outcomes

*Individuals* with intellectual and developmental disabilities and their families clearly articulated a set of meaningful outcomes that expressed a desire to be included and to feel good about their participation in community settings. Because participation, family quality of life, and well-being are individually defined and experienced, it is vital to engage individuals with intellectual and developmental disabilities and their families in these very early stages of research development.

*Stakeholders* wanted action and change to happen in their communities *now*, and often did not realize or understand that research “takes time.” Although there was acknowledgment that systemic change can be slow, and that progress may occur in small increments, there was a call to find ways to create connections and relationships and move forward. To address this call and the challenge of understanding that research does not always lead to immediate action or results, our Research Engagement Workgroup (Table 6) created a series of video training modules (Stakeholder Research Training Guides) that explain research as long-term change while inviting people to assess their readiness and willingness to engage in the type of change they would like to see in the long run.

*Organizations*, via our Communications and Engagement and Mentoring Network Workgroups (Table 6), expressed concern regarding where to find information and desired support for mentoring related to best practices for access and inclusive programming. There is potential for organizations that have been successful in this arena to serve and mentor other organizations. By resetting the culture and expectations within community programs and services, the diverse needs of individuals with disabilities are proactively considered. One participant noted, “If people within an organization know and interact regularly with people with disabilities, inclusion improves.”

### What We Learned About Multi-Stakeholder Engagement

Face-to-face meetings are a critical first step for engagement. People were clear: they wanted to be heard and they appreciated our approach, which brought the matrix of community members together. Our stakeholder groups were clearly motivated by opportunities to connect, network, and share information and resources with others with similar interests and priorities. This theme informs our efforts to expand and sustain engagement with stakeholder groups. Our face-to-face meetings broadly reflected a useful structure for facilitating engagement called community conversations (33, 34). We found that the structured but flexible approach of bringing diverse groups of stakeholders together and engaging them in complex topics involving local and systemic factors was a highly productive process. This approach provided an ideal structure for generating and prioritizing solutions, better understanding of what community participation meant within various community contexts and increasing agency of often marginalized stakeholders including individuals with disabilities and their family members.

Many of our stakeholders represented multiple roles including that of self-advocates also being family members. Accurately collecting and representing data from individuals with multiple roles was complicated. Importantly, we had individuals who represented both a healthcare professional and had a role within a community organization. This suggests that important partnerships and collaboration around inclusion and participation were occurring on multiple levels within some organizations and communities.

Communication that uses plain language, a variety of delivery modes, and accessible and usable materials is imperative. It was extremely useful to have our Lead Engagement Coordinator, who is autistic, review our stakeholder communications for plain language, use text that conveyed information briefly and concisely (e.g., bulleted lists), and attend to Flesch-Kincaid Reading Levels. Communication strategies, including the use of plain language, visual charts, and videos to gather and share information were effectively used by the project's Lead Engagement Coordinator to establish trust, safety, and belonging and to facilitate conversation among the different stakeholders.

Refining and improving communication is an important, ongoing process. In addition to print materials, we also presented information using video and audio so that stakeholders had access to a variety of information delivery modes. We first used video to help stakeholders prepare for our third regional meeting and found that it greatly enhanced accessibility. Participant feedback rated the video so highly that we used it again as part of our Year-2 invitation to join workgroups and as the format for the Stakeholder Research Training Guides. We learned of the importance of preparing materials with the assistance of experienced graphic designers and marketing experts. This information aids in the creation of appealing materials that will allow for dissemination of our content to a wide audience of consumers in our stakeholder communities. Our Advisory Board and workgroup members were excellent contributors in terms of identifying successful ways we communicated, as well as places or materials where communication improvements were needed. We believe the importance and impact of our intentional language and communication methods are best described by stakeholders who reviewed one of our Stakeholder Research Training Guides:

“I like that you used a simple metaphor, which is accessible to most people.”

“I like that the video uses plain language, instead of research jargon.”

“I like that this video is an invitation to do research.”

## Conclusion

Our long-term goal is to develop stakeholder-driven research that focuses on how participation interventions delivered in the community, and outside of healthcare settings, impact the health outcomes of individuals with intellectual and developmental disabilities and their families. This is important to emphasize because healthcare practitioners often apply or misapply medically based models in the community, and these approaches do not always translate into community settings or practice (35).

The overarching stakeholder research priority was “Measuring Success: When community organizations use so-called “best practices” for inclusive participation, how do we measure success?” This question anchors future research along with stakeholder-driven outcomes. Future research using multiple types of research methods is needed to address the various research priorities and patient-centered outcomes that were identified. For example, using a survey and qualitative research help us to understand the individualized experiences regarding very personal barriers, successes, and outcomes of participation in one's respective community, and may lead to an understanding of the population as a whole.

Our engagement process and activities may be suitable to generalize and transfer to other groups of individuals with intellectual and developmental disabilities or cognitive disabilities, children and youth with special healthcare needs, or individuals with chronic medical conditions. Principles of access, usability, and services that are universally designed to meet the widest range of user needs-characteristics of the community-level interventions this research team aims to explore-also can be intrinsically built into research planning and stakeholder engagement.

We believe there are several implications for policy that emerged from our conversations with stakeholders and key findings in relation to (1) civil rights and community participation; (2) education and transition; and (3) research participation and human subjects protection. The stakeholder engagement approach used in this study aligns with historical, current and emerging policies that aim to engage, educate, and empower individuals and families with information and resources, and promote inclusion and societal participation in all sectors of the community (18). Additionally, this study further informs the policy conversation around inclusion as a civil right of individuals with disabilities as evidenced by the Americans with Disabilities Act of 1990 and later by the Olmstead Decision of 1999 (36, 37). Our findings extend the conceptualization of inclusion outlined in these policies. Importantly, inclusion *in practice* is more than just allowing an individual access to get through the door. It will also be important to keep these priorities in mind in relation to future educational policy, such as potential reauthorization of the Individuals with Disabilities Education Improvement Act of 2004 (IDEA) (38), specifically with regard to provisions for transition planning and community-based programming to prepare youth for adulthood. As evidenced by our work, a wide range of community stakeholders can and should act as facilitators in the transition process by engaging youth to participate in all sectors of the community (e.g., social and leisure activities, inclusive employment and hiring and training practices) (7, 39). Clearly, there are also implications for research policy in terms of highlighting funding priorities as well as including people with intellectual and developmental disability as research partners. For example, being sensitive to balancing policies and procedures that allow for voices to be heard and support full participation as research stakeholders and partners, not just subjects, along with matters of protection for this population (40).

The examples of the engagement and communication methods we used and described are significant for including and ensuring that individuals with intellectual and developmental disabilities, a group underrepresented in research, can fully participate as stakeholders in research planning and processes. This is critical to move stakeholder driven research priorities forward in response to research initiatives that aim to address key issues around participation and health disparities for this population. Defining best practices for participation, particularly in developing research partnerships, however, continues to evolve, and is indeed an area that will benefit from diverse and collective strategies and sharing lessons learned as these strategies are implemented and expanded.

Although our project was not held during the COVID-19 pandemic, we acknowledge that community participation has changed and the need to expand these discussions strengthened. We have received a PCORI engagement award to expand our regional and national network, which will include the Central and Southwest United States. This will also allow us to clearly address the pandemic and post-pandemic participation with direct processes. We will continue this work with a focus in areas such as cultural and linguistic access and sustained stakeholder engagement and involvement in our future research activities.

## Data Availability Statement

The raw data supporting the conclusions of this article will be made available by the authors, without undue reservation.

## Author Contributions

All authors contributed to the development of the protocol, the stakeholder meetings, collection and analysis of data collected during the stakeholder meetings, drafting and editing of the manuscript and final approval of the manuscript for submission.

## Funding

The project was funded by the Patient Centered Outcomes Research Institute EAIN # 5964.

## Conflict of Interest

The authors declare that the research was conducted in the absence of any commercial or financial relationships that could be construed as a potential conflict of interest.

## Publisher's Note

All claims expressed in this article are solely those of the authors and do not necessarily represent those of their affiliated organizations, or those of the publisher, the editors and the reviewers. Any product that may be evaluated in this article, or claim that may be made by its manufacturer, is not guaranteed or endorsed by the publisher.
